# Detailed chemical analysis of honey bee (*Apis mellifera*) worker brood volatile profile from egg to emergence

**DOI:** 10.1371/journal.pone.0282120

**Published:** 2023-02-21

**Authors:** Amélie Noël, Charlène Dumas, Emilien Rottier, Dominique Beslay, Guy Costagliola, Christian Ginies, Florence Nicolè, Andrea Rau, Yves Le Conte, Fanny Mondet

**Affiliations:** 1 INRAE, UR 406 Abeilles et Environnement, Avignon, France; 2 INRAE, UR 1115 Plantes et Systèmes de Culture Horticoles, Avignon, France; 3 INRAE, UMR 408 Sécurité et Qualité des Produits d’Origine Végétale, Avignon, France; 4 Université de Lyon, UJM-Saint-Etienne, CNRS, LBVpam, Saint-Étienne, France; 5 INRAE, UMR 1313 Génétique Animale et Biologie Intégrative, Jouy-en-Josas, France; 6 BioEcoAgro Joint Research Unit, INRAE, Université de Liège, Université de Lille, Université de Picardie Jules Verne, Estrées-Mons, France; University of Alberta, CANADA

## Abstract

Chemical communication is a widely used mode of communication for social insects and has been demonstrated to be involved in many behaviours and physiological processes such as reproduction, nutrition or the fight against parasites and pathogens. In the honey bee, *Apis mellifera*, the release of chemical compounds by the brood plays a role in worker behaviour, physiology, and foraging activities and colony health as a whole. Several compounds have already been described as brood pheromones, such as components of the brood ester pheromone and (E)-β-ocimene. Several other compounds originating from diseased or varroa-infested brood cells have been described as triggering the hygienic behaviour of workers. So far, studies of brood emissions have focused on specific stages of development and little is known about the emission of volatile organic compounds by the brood. In this study, we investigate the semiochemical profile of worker honey bee brood during its whole developmental cycle, from egg to emergence, with a specific focus on volatile organic compounds. We describe variation in emissions of thirty-two volatile organic compounds between brood stages. We highlight candidate compounds that are particularly abundant in specific stages and discuss their potential biological significance.

## Introduction

Communication is fundamental to all life forms. Chemical communication, *i*.*e*. the emission of specific chemical compounds by an individual and received by another at a specific moment, is highly present in the living world. In eusocial insects, compounds emitted and perceived by individuals of the same species constitute a pheromonal message, which is part of the chemical language repertoire. Individuals receiving this chemical message will respond by adapting their behaviour or their physiology [1–3]. This type of communication is key to the regulation of superorganism homeostasis, as it allows eusocial species to organise the colony and the division of labour [4–6].

In the honey bee, *Apis mellifera*, chemical communication is widely deployed across castes and individuals [3]. It is well known that the brood communicates with the adults through the use of pheromones. An iconic example of brood chemical communication is the brood ester pheromone (BEP), composed of by a mixture of 10 fatty esters (methyl and ethyl oleate, methyl and ethyl linoleate, methyl and ethyl linolenate, methyl and ethyl palmitate, methyl and ethyl stearate) [7]. Another well-known brood pheromone is (E)-β-ocimene [8]. These two pheromones have both releaser (*i*.*e*. triggers an immediate behavioural response) and primer effects (*i*.*e*. changes the physiology of the receiver). As releaser effects, BEP triggers worker brood-rearing behaviour and cell-capping behaviour (closing brood cells with wax) [7, 9], and (E)-β-ocimene increases pollen foraging and brood-feeding behaviour [10, 11]. A common primer effect of these two pheromones is the inhibition of worker ovarian growth [8, 12].

Dead brood emit oleic acid and (E)-β-ocimene, which both trigger hygienic behaviour (detection of sick brood and cleaning of the brood cell), suggesting a death-signalling role for these two compounds [13]. Hygienic behaviour is also performed on diseased brood, before it dies. This is the case for brood infected by chalkbrood (*Ascosphaera apis*). In order not to become a vector of contamination, adults must detect brood parasitized by the fungus before the spores reach the cuticle of the larva. The hygienic behaviour is then triggered by the emission of phenethyl acetate, 2-phenylethanol, and benzyl alcohol [14]. A variant of hygienic behaviour, VSH (*Varroa Sensitive Hygiene*) behaviour, targets brood cells parasitized by the varroa mite and is triggered by compounds emitted by parasitized brood cells ((Z)-6-pentadecene, (Z)-10-tritriacontene, 6 VPS (*Varroa-Parasitization-Specific*: tricosan-2-one, pentacosan-2-one, tetracosyl acetate, heptacosan-2-one, hexacosyl acetate and nonacosan-2-one), α-pinene and ethyl hexanoate) [15–18]. Stressed or diseased brood emit other compounds but potential behavioural responses of workers have not been assayed. Such is the case for γ-octalactone, a compound that larvae infected with the European foulbrood emit at higher levels than healthy brood (*Melissococcus*. *plutonius*) [19]. *Varroa destructor* infestation and *Deformed Wing Virus* infection increase the emission of ten chemical compounds in brood targeted by hygienic behaviour (pentacosane, hexacosane, 4-methyltetracosane, 9- and 11-methyltricosane, 11- et 13-methylpentacosane, 12- et 14-methylhexacosane, pentacosene, heptacosene, hentriacontene) [20]. With the exception of a few volatile organic compounds (VOC), the majority of chemical compounds already described as brood emissions that trigger adult behaviours are not very volatile. Little is known about highly volatile compounds of honey bee brood; studies have tended to focus on low-volatile compounds. Unlike cuticular hydrocarbons, little is known about VOC emission of brood in the other social insect [4, 21, 22]. The role of the brood on the behaviour (e.g. foraging regulation) and physiology (e.g. ovary maturation) of adults in social insects is well established. For example, in ants, brood has been shown to have a role in adult physiology and behaviour, but identification of this potential ant brood pheromone is still lacking [see review: 23]. In bumblebees, the pupal odour attracts the workers but has no impact on the physiology of the adult. The concept of a brood pheromone in the bumblebee is still discussed [24].

The emission of highly volatile chemical compounds by the brood allows information to be disseminated quickly and over a long distance within the nest. Studying emission of VOC by the brood would provide a better understanding of its chemical communication and development.

Thus far, no documentation of VOC emissions over the entire brood development is available, as studies describing VOC emissions from brood focus on specific stages of development and/or on specific compounds [25–27]. Maisonnasse *et al*. [27] showed the difference in (E)-β-ocimene emission between larvae and pupae (first larval stage to fifth pupal stage). Carroll & Duehl [25] carried out *in situ* VOC captures on unstressed brood from hatching to the pre-pupal stage, in the presence of nurses on the frame. The authors showed changes in the emission of six VOC from the brood according to age (2-heptanone, 3-carene, β-ocimene, methyl benzoate, octanoic acid and decanal) and reported the presence of 22 VOC in total (2-heptanol, 2-heptanone, 2-pentanone, 3-carene, 3-methyl-1-butanol, 6-methyl-5-hepten-2-one, α-pinene, citral, decanal, decane, ethyl benzoate, (E)-β-ocimene, geraniol, heptanal, hexanal, isobutyl acetate, isopentyl acetate, methyl benzoate, nerol, nonanal, octanal and nonane) [25]. Light *et al*. [26] also captured VOC *in situ* emitted by worker and drone brood at different developmental stages (last day of egg, first instar larvae, first, third and fifth instar pupae for drone brood, fifth instar pupae for cold-killed drone brood, fifth instar pupae for worker brood). They identified 75 VOC emitted by worker and drone brood frames that triggered an electrophysiological response in *V*. *destructor*. These different studies give an indication as to the VOC emitted by bee brood but only provide a fragmented view of VOC emissions during its development.

Similarly to "-*omic*" studies (genomics, transcriptomics, proteomics, metabolomics and metagenomics), capturing VOC emissions of the brood throughout its development, from oviposition to emergence, makes it possible to observe and capture the essence of brood-adult chemical communication in its entirety. Studying VOC emissions has led to a better understanding of reproduction in beetles [28] and the estimation of larval and pupal age in blowflies [29, 30]. In honey bees, VOC collection permits the study of the changes in larval food consumption and pheromonal emission over the course of their development [25, 27].

Bee brood development is characterised by a sequence of different physiological stages. The brood passes from an open brood state comprising the egg stage and five larval stages, to a capped brood state (*i*.*e*. brood cells closed with a wax cap by workers) before the emergence of the adult bee, comprising a sealed larval stage, a prepupal stage, and nine pupal stages [6]. Physiological changes in the brood can be observed through the brood haemolymph proteome [31, 32]. These important physiological changes can also be observed in brood-nurse interactions according to the developmental stage. Under the influence of brood pheromones, workers adapt the food they give to the brood according to the age of the larvae [9, 33, 34], cap brood [7], modify their foraging behaviour [10, 35], and realise hygienic behaviour [13–17].

In this study, we develop an *in situ* experimental approach to analyse VOC emissions of healthy worker brood during the whole developmental cycle (21 days, from egg stage to adult bee emergence). Our target is to highlight the temporal changes in brood-characterising compound presence and abundance during the entire brood development. We highlight semiochemical compounds emitted by brood that may have a role in the communication between brood and workers.

## Materials and methods

### Honey bee colonies and experimental setup

This study was performed with nine local honey bee colonies (*A*. *mellifera*) installed at the INRAE (Institut National de Recherche pour l’Agriculture, l’Alimentation et l’Environnement) research centre in Avignon (France). As the aim of this study was to capture volatile organic compounds (VOC) in healthy brood, we were very careful to use colonies that were free of parasites and disease. The health of these colonies was closely monitored throughout the experiment: colonies were qualitatively screened by our beekeepers to confirm the absence of brood illness. This study was conducted from April to July 2021, when varroa infestation is at its lowest, to maximize chances that capped brood cells were not infested by the parasite.

**Fig 1:**
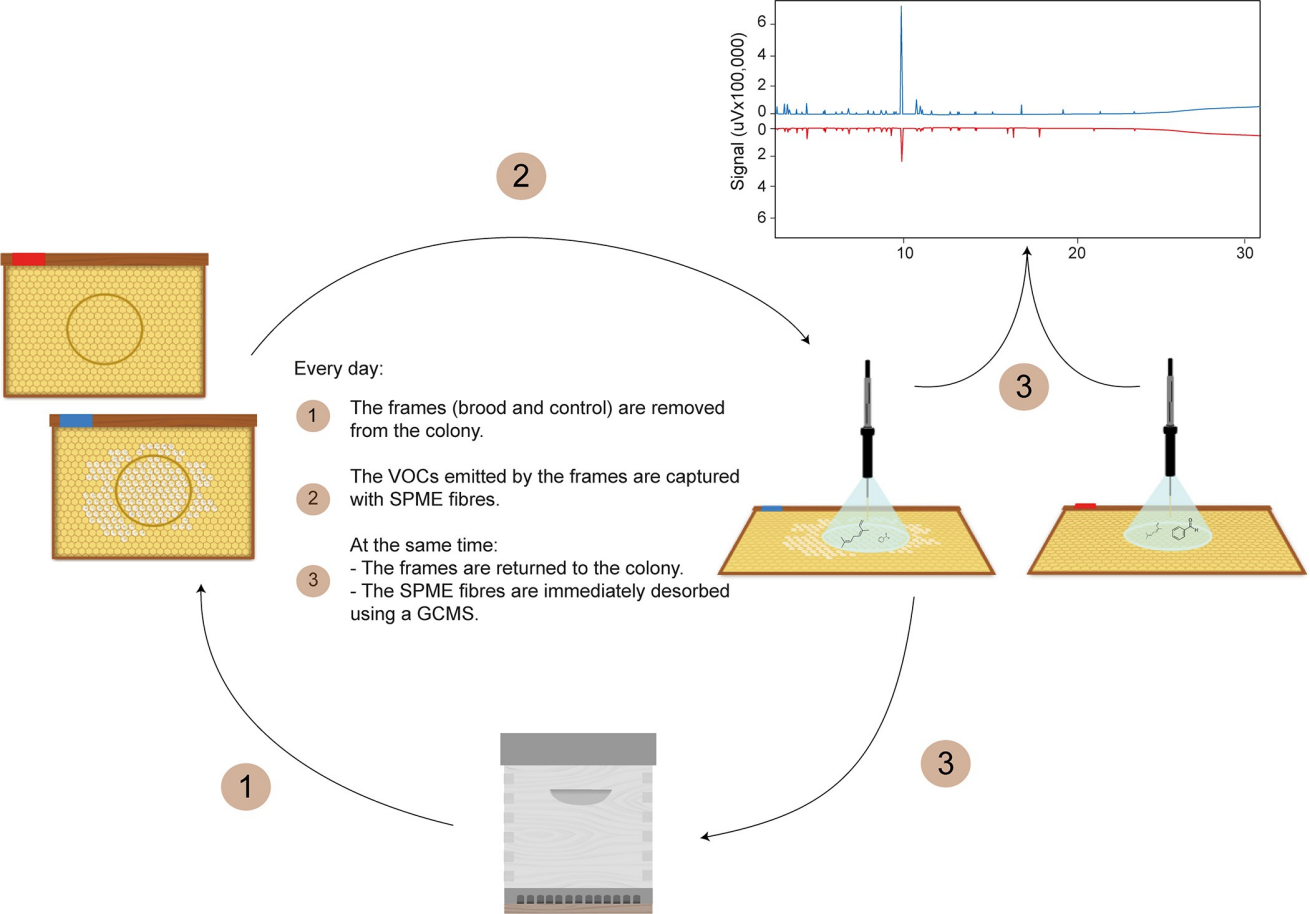
Experimental setup used for the *in situ* capture of the volatiles organic compounds (VOC) during honey bee brood development. Every day for 21 days, brood and control frames were removed from their colonies. VOC of the same patch of cells (brood and control) were captured for 20 minutes with a SPME fibre, in a room with controlled temperature and humidity (34–35°C, 65% RH). After capture, frames were immediately put back in their colony while fibres were desorbed on a GC-MS. Nine colonies were used, each with a control and a brood frame (371 captures in total).

To ensure working with brood of a homogeneous age, queens of the nine selected colonies were caged on an empty frame for 24h to allow for egg laying. After that period, the queen was removed from the cage and no longer allowed access to the laid frame (*brood frame*). An empty built frame (no brood, pollen or nectar) was concomitantly used as a control in each colony (*control frame*). On the first day of the experiment (following queen laying), circles containing about 130 cells were drawn on both brood and control frames to select cell patches to be monitored daily, for 21 days. Each day, brood and control frames were removed from their colonies and brought to the laboratory for chemical analysis. Experimental procedures were optimised to minimise any source of stress. To do so, adult bees were gently removed from the brood frame with a soft brush, and the brood was placed directly in a warm, moist box during the short journey from the hives to the experimental room. They were kept in a room with controlled temperature and humidity (34–35°C, 65% RH). The same patch of cells was monitored each day (Fig 1), and the content of each cell was recorded on a transparent plastic sheet.

### Volatile organic compound capture and analysis

VOC capture was conducted passively, using a polydimethylsiloxane divinylbenzene (PDMS DVB) Solid Phase Micro Extraction (SPME) fibre (65 μm, manual holder). The fibre was inserted inside a glass funnel (Ø 70 mm) placed upside down on the patch of cells to be monitored (Fig 1). For each capture, the funnel was pressed against the waxy surface to form a hermetic junction between the sample (cell patch) and the capture system. Captures lasted for 20min, after which frames were returned to their colonies. Each capture of a brood sample was paired with that of a control sample (seven control samples could not be captured due to bad weather conditions on day 11 and 12), thus forming a total of 371 captures (9 control + 9 brood samples per day). Qualitative and quantitative analyses of VOC were performed on a gas chromatograph coupled with mass spectrometry (GC-MS). SPME fibres were desorbed into a GC-MS (Shimadzu GCMS-QP2010SE), equipped with an electronic impact ion source (70 eV) and a ZB 5 MS column (Zebron ZB 5ms, 20 m x 0.18 mm, 0.18 μm thickness). Desorption was realised in splitless mode, with the constant column flow at 0.82 mL/min; the carrier gas was helium. The injector temperature was set at 250°C and the oven temperature program as follows: 45°C isothermal held for 5 min, followed by temperature increases at a rate of 10°C/min up to 250°C. The oven was finally held at 250°C for 4 min. The ion source was set at 200°C and m/z scanned from 40 to 350 amu. Compound identification was performed by comparison of their mass spectra and retention indices with those found in the NIST 2017 library (v2.3). This identification was further confirmed by injection of the reference standards purchased from Sigma Aldrich (except for hexenyl acetate (s24) for which the location of the unsaturation remains unknown).

### Statistical analyses

Statistical analyses were performed using R (version 4.0.5). To account for non-linear trends across time, generalized additive mixed models (GAMM) were fit on total peak area for each day and each sample group (brood, control) to investigate differences between control and brood samples and their evolution over time (R package “mgcv” v1.8–39; https://cran.r-project.org/web/packages/mgcv/index.html [36]).

Three redundancy analyses (RDA) were performed on three different phases of development, based on GAMM results (“Eggs”: day 1 to day 3; “Larvae and Non-Sclerotised Capped Brood”: day 4 to day 16; “Sclerotised Pupae”: day 17 to day 21), to identify compounds that characterise the brood (R package “vegan” v2.6–2; https://cran.r-project.org/web/packages/vegan/index.html). Day and sample group (brood and control) and their interaction were included as fixed factors in the RDA to identify brood-characterising compounds. To graphically represent the RDAs, we selected the two components which explain the largest proportion of the data variance. Compounds detected in at least five out of the nine replicates in brood samples for any given day were included in the analyses. From the 3 RDAs, a list of 32 brood-characterising compounds was compiled. We then split brood development into eight characteristic periods to study the compounds or chemical families that characterised each period, as well as those that covaried together. The four days around the capping of brood cells by the workers, represent a key moment in the development of the brood, both physiologically and behaviourally [3, 6, 7, 37]. For this reason, those four days were separated into four one-day periods. The eight periods were thus defined as follows: Eggs: days 1–3; 1^st^ to 4^th^ instar larvae (L1-L4): days 4–7; 5^th^ instar larvae (L5): day 8; Capping: day 9; Sealed larvae (SL): day 10; Prepupae (PP): day 11; Non-sclerotised pupae (NSP): day 12–16; and Sclerotised pupae (SP): days 17–21.

In order to classify the data, we chose to group the compounds found according to their respective chemical families. We chose this presentation not because of the potential effects on behaviour, but rather to highlight a classification based on the chemical structure of these molecules, in particular to evaluate shared patterns of variation and stage-specific emission.

As our target is to highlight the temporal changes in the presence and abundance of brood-characterising compounds during development, we normalised brood compound area values by those measured in the control (*i*.*e*. (A_brood_—A_control_)/A_control_) for each day, compound, and colony. To avoid division by zero, a constant ten times lower than the smallest non-zero value was added to the data. We then calculated the median normalised area of each compound across the nine colonies for each day. Negative median values, indicating a higher amount of the compound in control samples than in brood samples, were set at zero. Compounds were subsequently quantified relative to the highest median normalised value observed across the 21 capture days. We represented these relative median normalized values in a heatmap with a log_10_ scale (adding a constant of 1). We similarly calculated normalised brood compound area by stage, analogous to that done by day. For two compounds, these stage-specific median normalised values were equal to 0 as they were observed on a single day of the four days comprising the SP stage. As before, compounds were quantified relative to the highest median value observed across the 8 developmental stages. The evolution across stages of relative median normalised compound emissions was visualized in another heatmap. We realised a clustering of the arcsine-transformed relative median normalised values per stage using a Gaussian mixture model to highlight compounds that shared stage-specific maximum emissions (R package “coseq” version 1.17.2; https://bioconductor.org/packages/release/bioc/html/coseq.html [38]). The number of clusters was identified based on the integrated completed likelihood (ICL) model selection criterion.

For comparisons with the scientific literature, we calculated the estimated emission per mg of individuals weight for (E)-β-ocimene. As the tracking of the individuals throughout the experiment did not allow us to weigh the brood, we instead used in-house brood weight data (first larval to fifth pupal stage, day 4 to day 15 in this paper, n = 9 per stage) obtained in a previous experiment on similar colonies.

## Results

### Total compound emission profile across development

**Fig 2:**
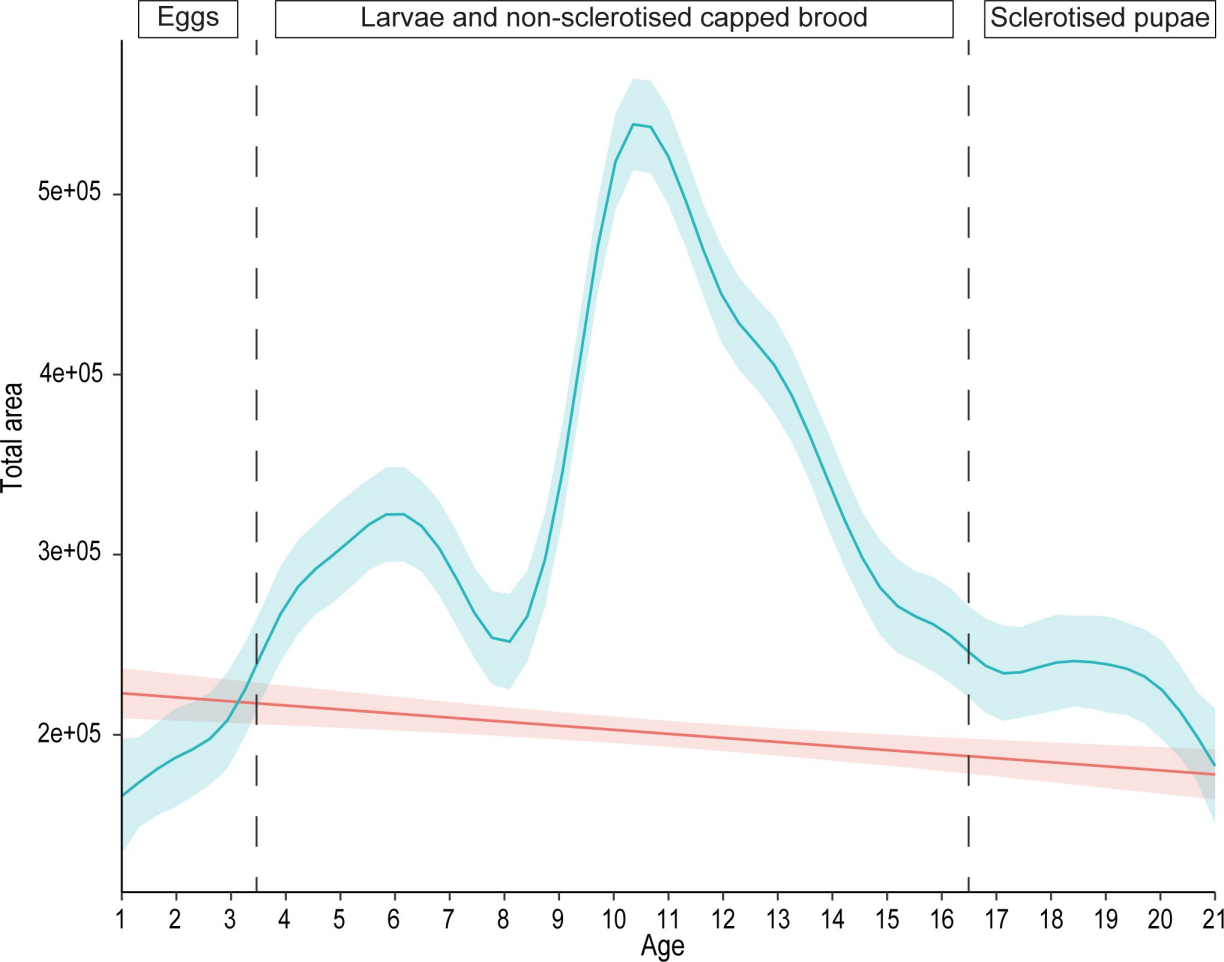
Evolution of the total peak area of all compounds across development (GAMM models). Lines show the mean total area and the surrounding 95% standard error, for brood (blue) and control (red) samples. Dotted lines separate the three groups: “Eggs” (day 1 to 3), “Larvae and non-sclerotised capped brood” (day 4 to 16) and “Sclerotised Pupae” (day 17 to 21) considered for the multivariate analyses.

A comparison of total peak area between control and brood samples revealed that all compounds (total peak area) are significantly more abundant in brood samples than in control samples (GAMM; Brood: estimate = 296,010, p < 2e^-16^; control: estimate = 200,479, p < 2e^-16^) (Fig 2). The quantity of compounds fluctuates significantly from 2.6e^5^ (day 16) to 5.4e^5^ (day 11) of total compound area (GAMM; Brood over time: F = 12.403, p < 2e^-16^; Control over time: F = 0.078, p = 0.820) (Fig 2). The first three days (egg stage) and the last five days (sclerotised pupal stages) are periods of low overall emissions, with small differences between brood and control samples (Fig 2). From day 4 (first instar larvae) to day 16 (fourth instar pupae), total compound abundance is quite variable and strikingly different between brood and control samples, with a notable peak of brood emission around days 10 and 11 (prepupae) (Fig 2). Based on these tendencies, and to improve the brood-characterising compound determination step, we separated our dataset into three groups to perform multivariate redundancy analyses (RDA): Egg (day 1 to day 3), Larvae and Non-Sclerotised Capped Brood (day 4 to day 16) and Sclerotised Pupae (day 17 to day 21).

### Identification of brood-characterising compounds

Based on the total emission analysis and the identification of 3 distinct phases of compound emission, we performed three redundancy analyses (RDAs).

**Fig 3:**
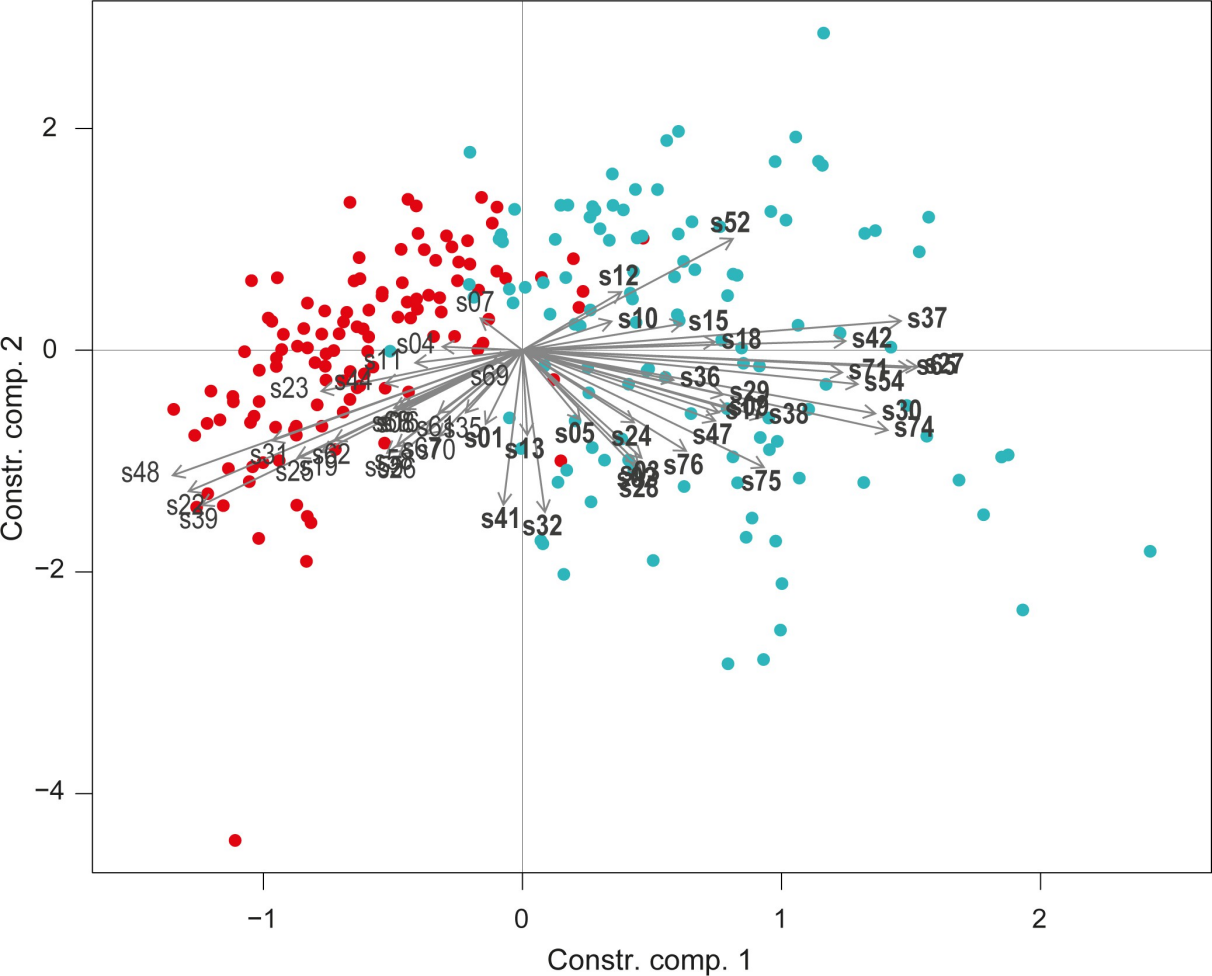
Redundancy analysis (RDA) performed on chemical compounds captured on brood and control samples for the “larvae and non-sclerotised capped brood” group (day 5 to day 16). For each day: brood sample n = 9, control sample n = 9. Each dot represents a sample (blue: brood, red: control). Compounds are represented by their IDs and grey arrows. The compounds characterising the brood and retained for the rest of the analysis are shown in bold.

In the largest RDA (day 4 to day 16, “Larvae and non-sclerotised capped brood” group), the first two components of the RDA explained 56.82% of the variance. Both day and sample group, as well as their interaction, were significant (Table 1). The two sample groups (brood and control) were well separated on the first component (Fig 3). Based on this analysis, twenty-seven compounds characterising the brood samples were retained (s01, s02, s03, s05, s09, s12, s13, s15, s17, s18, s24, s27, s28, s29, s30, s32, s36, s37, s38, s41, s42, s47, s52, s54, s65, s71, s74, s75, s76) (Fig 3, Table 2).

**Table 1:** Results of the 3 RDAs showing the effects of day and sample groups on compound area. For each day: brood sample n = 9, control sample n = 9.

Groups	Factors	F	P
Eggs (*day 1 to day 3*)	Day	2.04	0.008
	Sample group	4.39	0.002
	Day: Sample group	0.49	0.994
Larvae and non-sclerotised capped brood (*day 4 to day 16*)	Day	2.34	0.001
	Sample group	22.92	0.001
	Day: Sample group	1.45	0.001
Sclerotised Pupae (*day 17 to day 21*)	Day	1.89	0.001
	Sample group	7.28	0.001
	Day: Sample group	0.73	0.950

**Table 2:** List of chemical compounds found as brood-characteristic during brood development (21 days). Days of detection represent periods where a given compound is found in at least 5 replicates in brood samples for a given day. *: Unsaturation remains unknown, **: Stereochemistry remains unknown.

ID	Compounds	Chemical families	Retention time	Retention index	Days of detection
s01	3-Methyl-2-buten-1-ol (Prenyl alcohol)	Alcohol	2.947	784	4–6, 9
s02	2,3-Butanediol ** (diastereomer)	Alcohol	3.112	789	8–16, 18–20
s03	2,3-Butanediol ** (diastereomer)	Alcohol	3.307	795	8–16, 18–20
s05	Octane	Alkane	3.447	800	3–5, 8–11, 14–21
s09	1-Hexanol	Alcohol	5.473	869	8–21
s10	Methylbutyl acetate (Isoamyl acetate)	Ester	5.735	877	1, 4–8, 10–12, 14, 16, 19–20
s12	2-Heptanone	Ketone	6.090	890	4–9
s13	Nonane	Alkane	6.435	900	5, 9–10, 13, 16–19
s15	α-Pinene	Terpene	7.273	934	2–9, 11–20
s17	1-Heptanol	Alcohol	8.260	973	12, 16, 18, 21
s18	β-Pinene	Terpene	8.345	977	3–4, 6, 11–20
s24	2-Hexenyl acetate **	Ester	9.385	1023	11
s27	(Z)-β-ocimene	Terpene	9.660	1036	6–18
s28	Phenylacetaldehyde	Aldehyde	9.750	1042	6, 9–13, 15, 17–21
s29	α-Ocimene	Terpene	9.800	1045	7, 11, 15, 17
s30	(E)-β-Ocimene	Terpene	9.886	1047	1–21
s32	1-Octanol	Alcohol	10.348	1072	9–20
s35	(E)-Linalool furanoxide	Alcohol	10.623	1086	19
s36	2-Nonanone	Ketone	10.713	1090	6–9
s37	Methyl benzoate	Ester	10.753	1092	1–21
s38	Linalool **	Alcohol	10.89	1099	9–14, 16–20
s41	Phenylethyl alcohol	Alcohol	11.085	1111	9–12, 18–20
s42	Allo-ocimene	Terpene	11.368	1127	6–7, 10–16
s45	(E)-Linalool pyranoxide	Alcohol	12.080	1170	19
s47	Methyl salicylate	Ester	12.428	1190	4–12, 14–16, 18–19
s52	γ-Octalactone	Ester	13.403	1253	1–15
s54	Tridecane	Alkane	14.105	1300	1, 5–21
s65	Pentadecane	Alkane	16.793	1500	4–21
s71	Heptadecane	Alkane	19.172	1700	1, 4–18
s73	Nonadecene *	Terpene	21.087	1880	19–21
s74	Nonadecane	Alkane	21.315	1900	4–21
s75	Eicosane	Alkane	23.262	2000	8–21

In the “Egg” RDA analysis (days 1–3), the first two components of the RDA explained 73.95% of the variance. Both day and sample group were significant (Table 1), but the interaction was not, confirming that overall brood and control compound emissions maintain the same temporal trend. Both sample groups (brood and control) were also well separated on the first component (S1 Fig). Five compounds characterising brood samples, all included in the previous list, were highlighted by the RDA (s15, s18, s30, s37, s52) (S1 Fig, Table 2).

In the “Sclerotised pupae” RDA (day 17 to day 21), the first two components explained 64.07% of the variance. Both single factors were significant, but their interaction was not (Table 1). The two sample groups were once again well separated on the first component (S1 Fig). Twenty-eight compounds characterising the brood sample were retained, partially overlapping with the previous lists (s01, s02, s03, s05, s09, s10, s12, s13, s15, s17, s18, s27, s28, s30, s32, s35, s37, s38, s41, s45, s47, s54, s65, s71, s73, s74, s75, s76) (S1 Fig, Table 2).

Using these three RDAs, we were able to compile a final list of 32 VOC characterising the brood samples (S2 Fig, Table 2).

### Evolution of brood-characterising compound emission during brood development

**Fig 4:**
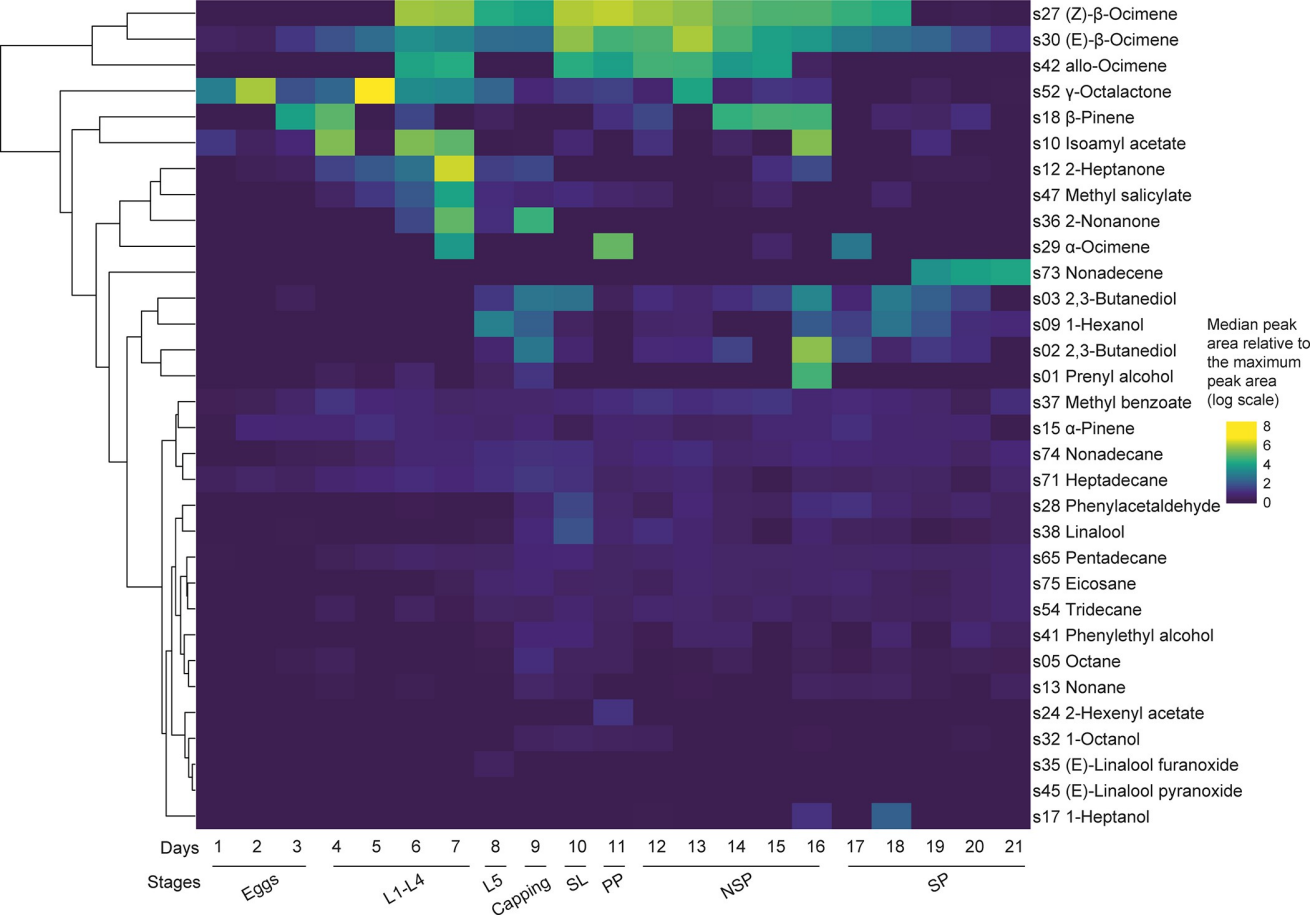
Heatmap showing the temporal evolution of the amount of the 32 compounds characterising brood throughout development. The temporal evolution of each compound area is represented in rows, with a colour gradient representing the median normalized peak area relative to the respective maximum for each compound (in log scale). The colour change represents a change in emission of the compound, with a maximum emission in yellow and a minimum in dark blue. Each column represents the age of brood development. Compounds are labelled with their IDs and chemical names. A dendrogram representing a hierarchical clustering (Euclidean distance, complete linkage) of compounds is indicated on the left.

Each day of brood development was characterised by different emission of VOC. The 32 VOC could be classified into two groups; a group of compounds with a marked variation of emission over time and a group with a more constant emission. The group of compounds fluctuating over time included 15 compounds (in order of appearance in the Fig 4, from top to bottom: s27, s30, s42, s52, s18, s10, s12, s47, s36, s29, s73, s03, s09, s02 and s01) (Fig 4). Except for α-pinene (s15), all terpenes found in this study were in this group (s27, s30, s42, s18, s29 and s73, Fig 4). The same pattern applied to the two ketones (s12 and s36, Fig 4).

The group of compounds with a more constant emission over time included 17 compounds (s37, s15, s74, s71, s28, s38, s65, s75, s54, s41, s05, s13, s24, s32, s35, s45 and s17) (Fig 4). The seven alkanes found in this study were in this group (s74, s71, s65, s75, s54, s05 and s13), as well as the aldehyde (s28) (Fig 4). For the alcohols and esters found in this study, their fluctuation over time depends on the compound. Four alcohols showed a marked change over time (s03, s09, s02 and s01), and six others displayed more constant emissions level (s38, s41, s32, s35, s45 and s17) (Fig 4). Two esters fluctuated strongly over time (s52, s10 and s47) and two others remained constant (s37 and s24) (Fig 4).

**Fig 5:**
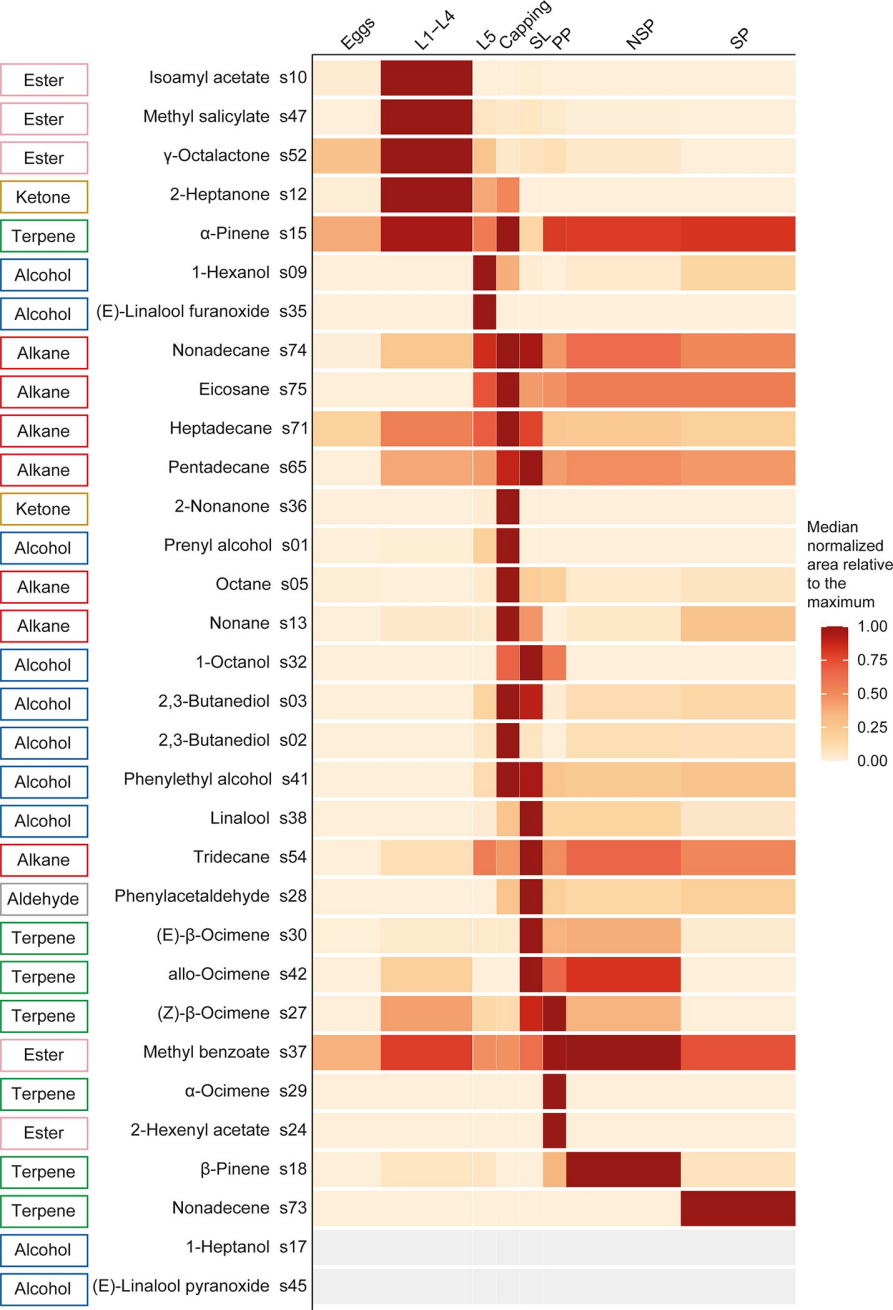
Heatmap showing the temporal evolution of the relative amount of the 32 brood characterising compounds, along developmental stages. The evolution of the quantity of each compound is represented in rows, with a colour gradient. The absence of compounds is represented in grey (here, both compounds are present only on a single day of the SP stage, resulting in a median of 0 for all stages). Each column represents a key stage of brood development (Eggs: day 1 to day 3; L1-L4: 1^st^ to 4^th^ instar larvae, day 4 to day 7; L5: 5^th^ instar larvae, day 8; Capping: adult capping behaviour, day 9; LS: larvae spinning cocoon, day 10; PP: pre-pupae, day 11; NSP: non-sclerotised pupae, day 12 to day 16; SP: sclerotised pupae, day 17 to day 21). The width of each box is proportional to the duration of each stage. Compounds are labelled with their IDs, chemical names and chemical families.

To study the compounds or chemical families that characterised each brood stage as well as those that covaried together, the 21 days of brood development have been divided into eight periods, as follow: Eggs: days 1–3; 1^st^ to 4^th^ instar larvae (L1-L4): days 4–7; 5^th^ instar larvae (L5): day 8; Capping: day 9; Sealed larvae (SL): day 10, Prepupae (PP): day 11, Non-sclerotised pupae (NSP): days 12–16, Sclerotised pupae (SP): days 17–21. Different compounds were characteristic of the eight brood developmental stages (Fig 5). None of the compounds had its maximum at the egg stage (days 1–3), but six compounds were found in greater quantities in brood containing eggs than in the control (s15, s18, s30, s37, s52 and s71) (Fig 5 and S1 Fig). None of these six compounds were specific to the eggs; their emission increased upon hatching. In the first larval instars (L1-L4, days 4–7), four compounds reached their maximum emission (s10, s47, s52 and s12), and two other compounds neared it (s15 and s37) (Fig 5). In the last larval stage (L5, day 8), the blend began to change drastically. Compounds with their greatest quantity in the L1-L4 stage decreased (s10, s47, s52, s12 and s37), while the quantity of other compounds began to increase (s09, s35, s74, s75, s71 and s54) (Fig 5). During the capping period (Capping, day 9), 11 compounds had their maximum emission peak (s15, s74, s75, s71, s36, s01, s05, s13, s03, s02 and s41) (Fig 5). All of these compounds decreased during the sealed larval stages (SL, day 10) (Fig 5). In this stage, seven compounds had their maximum emission peak (S65, s32, s38, s54, s28, s30 and s42) (Fig 5). In the prepupal stage (PP, day 11), only three compounds reached their maximum emission peaks (s27, s29 and s24) and two compounds were slightly under maximum emission levels (s15 and s37) (Fig 5). In the non-sclerotised pupal stage (NSP, days 12–16), two compounds had their maximum emission peaks (s37 and s18) and two others were slightly under maximum emission levels (s15 and s42) (Fig 5). In the last stage of brood development (SP, days 17–21), only one compound was at its maximum (s73) (Fig 5).

The total amount of (E)-β-ocimene (s30) was higher during capped brood stages (SL, PP, NSP and SP) than in larvae, especially just after capping. When the amount of (E)-β-ocimene (s30) was reported per mg of individual weight, its quantity was found to be higher in larval brood stages than in capped brood stages and reached its maximum in first instar larvae (day 4) (S3 Fig).

**Fig 6:**
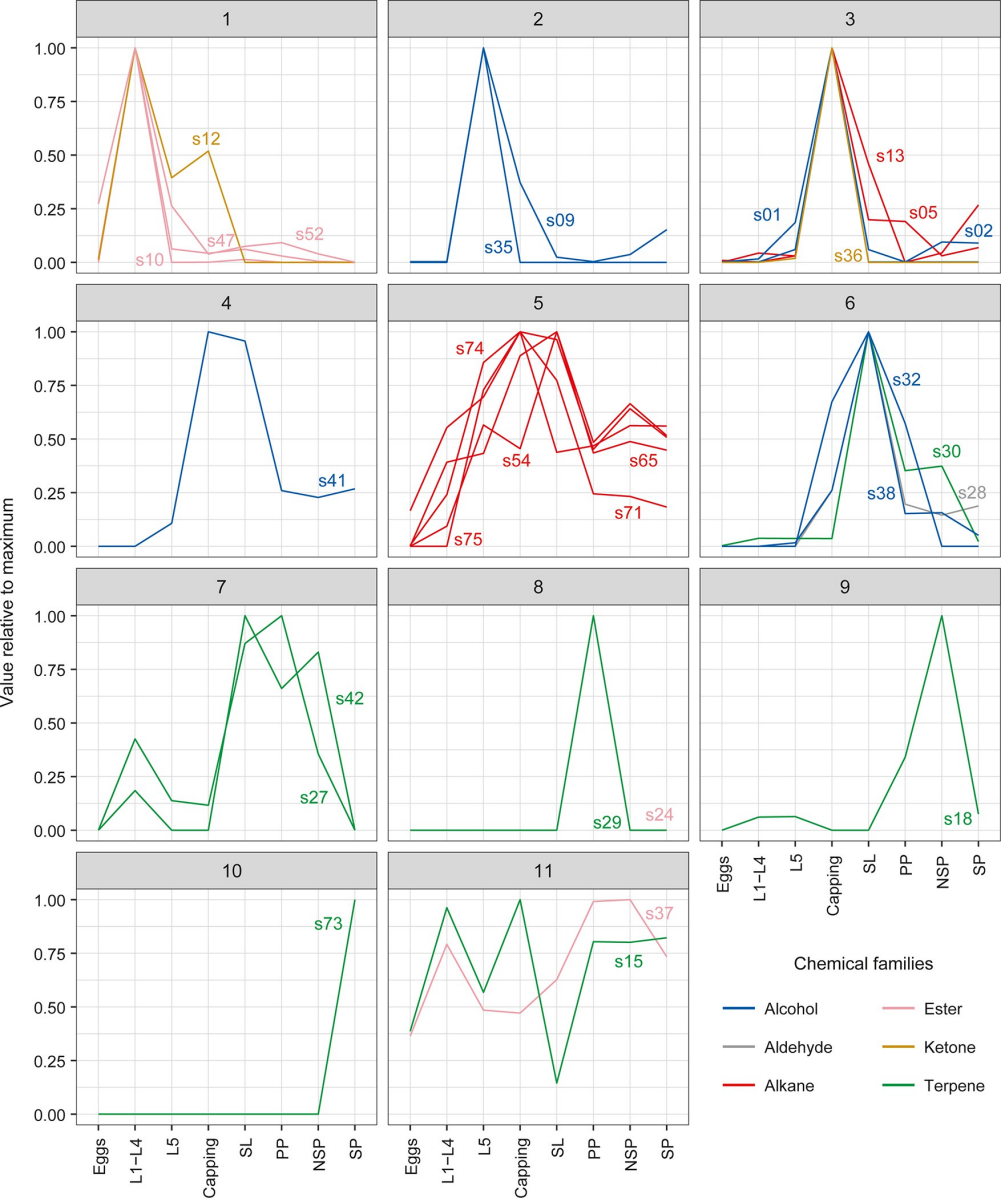
Clusters (Gaussian mixture models) showing the temporal variation of the relative (with respect to the maximum across stages) median normalized amount of the 32 brood-characterising compounds along developmental stages. Clusters group compounds with similarly timed emission peaks. Compounds are labelled with their IDs and coloured according to their chemical families. Eggs: day 1 to day 3; L1-L4: 1^st^ to 4^th^ instar larvae, day 4 to day 7; L5: 5^th^ instar larvae, day 8; Capping: adult capping behaviour, day 9, LS: larvae spinning cocoon, day 10, PP: pre-pupae, day 11, NSP: non-sclerotised pupae, day 12 to day 16; SP: sclerotised pupae, day 17 to day 21.

Depending on their chemical families, the compounds highlighted in this study did not have their maximum emission peaks at the same stages of brood development. Ketones had their maximum emission peak at L1-L4 for s12, and Capping stages for s36 (clusters 1 and 3, Fig 6). Except for s37, which had its maximum peak at the NSP stage, and s24, which had its maximum peak at PP stage, the esters had their maximum emission peaks at the L1-L4 stage (Fig 6). The alcohols peaked at the late larval stages (L5, Capping and SL) (clusters 2, 4 and 6, Fig 6). Alkanes had their peaks at the capping and just after (Capping and SL) (clusters 3 and 5, Fig 6). The aldehyde had its peak at the SL stage (cluster 6, Fig 6). Finally, except for s15 which had a double peak at L1-L4 and Capping (cluster 11), all of the terpenes had their maximum emission peaks after capping of the brood cells (clusters 6, 7, 8, 9, 10 and 11, Fig 6).

## Discussion

In this study, we analysed the VOC emitted by honey bee brood (*A*. *mellifera*) throughout its development. We identified 32 VOC characterising the brood, belonging to six different chemical families. The compounds did not have the same temporal dynamic during brood development and most of them appear to be stage-dependant. Chemical families were also specific to developmental stages.

Headspace capture is a common method for studying volatile emissions from bee brood [8, 13, 16, 27, 39, 40]. The focus of our study is to describe the landscape of chemical emissions from bee brood so that it can serve as a base for future, more functional studies. Some compounds were not fully identified (e.g. s24–2-Hexenyl acetate for which its stereochemistry is not found or s73 –Nonadecene where the location of the double bond is still unknown) and would require further identification.

To date, this study presents for the first time the emission of VOC from honey bee brood during its whole development, from egg laying to emergence. Some previous studies showed the evolution of specific compounds during the larval or early pupal stages [25, 27] or the presence of compounds in larvae or pupae at specific ages [26, 41]. A key point of our method was the monitoring of the same brood cells throughout development, with a non-invasive passive capture. No food resources (pollen, nectar, honey) were present on the brood frames, except for the larval jelly necessary for larval nutrition, which suggest that the compounds retained in this study are representative of the brood. Of the 32 compounds described in this study, 15 compounds are newly described in bees, with one compound never described either in bees or in the hive environment but has been described in floral emissions [42], and 14 of them already described as being present in the hive environment (wax, propolis, pollen, honey, ambient air) [43–48]. The other 17 compounds had already been described in bees [8, 19, 26, 40, 41, 49–55], with 13 already described has emitted by bee brood [8, 19, 26, 40, 41, 51, 52, 55].

### Ocimene brood emission

(E)-β-ocimene (s30) was the compound that was captured in the greatest quantity throughout this experiment, four times higher than the other compounds. Thus, the total peak area of the full set of compounds largely reflected the dynamics of (E)-β-ocimene (s30) during brood development. (E)-β-ocimene (s30) was found every day during brood development, from the first day of the egg stage to the emergence of the adult bee. This compound has already been described in the scientific literature in the uncapped brood to early capped brood stages [25, 27]. Its presence in the emission of VOC from eggs is described here for the first time. In our study, (E)-β-ocimene (s30) is more abundant in the capped stages than in the uncapped larval stages, with a peak in the SL stage. However, when the amount of compound is calculated according to the weight of the individuals, the peak of (E)-β-ocimene (s30) appears to be on day 4, at the first instar larvae. As a consequence, the amount of (E)-β-ocimene (s30) emitted by an individual is higher in the uncapped larval stages, especially in early larval stages, than in the capped brood stages. These results are in accordance with the results obtained by Maisonnasse *et al*. [27], thus providing additional validation to our experimental set-up. The presence of this compound in stages other than uncapped and early-capped brood raises questions about its possible role in other stages of brood development and its involvement in chemical communication between the brood and the adults of the colony.

In addition to (E)-β-ocimene (s30), we identified three ocimene isomers: (Z)-β-ocimene (s27), α-ocimene (s29) and allo-ocimene (s42). Allo-ocimene (s42) was previously described as a putative pheromone emitted by young larvae, in a similar context to (E)-β-ocimene [55]. Here, we found the presence of allo-ocimene in more than five of our samples mainly in the 3^rd^ and 4^th^ instar larvae as well as from the SL stage to the end of the NSP stage. Similarly to (E)-β-ocimene, allo-ocimene triggers a rapid nervous response of the antenna [55]. In addition, the ocimene is processed by the antennal lobe [56], the first brain region processing olfactory information sent by the antennal neurons [57]. Neither of the two other ocimenes captured in this study have been described as part of brood pheromones. Nevertheless, the response of adults to (E)-β-ocimene and allo-ocimene, and the high similarity between these four ocimenes, suggest that nurses may detect (Z)-β-ocimene and α-ocimene. Like (E)-β-ocimene with the BEP, the emission of these three other ocimenes could have an additive or synergistic effect and trigger different behaviours in adults [10, 27]. Further studies on behaviour and brain processing of olfactory information of nurse bees are necessary to highlight the potential discrimination between these four ocimenes, and the potential role of the (Z)-β-ocimene, the α-ocimene and the allo-ocimene.

### Compounds known to transmit pheromonal information between adult bees

In our study, we found four compounds (1-hexanol (s09), isoamyl acetate (s10), 1-octanol (s32) and 2-heptanone (s12)) already described as part of pheromone alarm mixtures emitted and received by adult bees [50, 53, 54, 58]. The alarm pheromone is composed of a cocktail of nearly twenty chemical compounds [3, 54, 59].

The compound 2-heptanone is known to be synthetized by the mandibular glands [53, 58]. During the larval stage, nurses feed the brood with royal jelly for the first stages and then with a mixture of pollen, honey and glandular extract from the mandibular and hypopharyngeal glands [60, 61]. The compound 2-heptanone has been described as a royal jelly VOC [45] and from VOC of uncapped brood frames in the presence of adult workers [25]. In our study, 2-heptanone (s12) was present in all uncapped larval stages, with a peak in 4th instar larvae (L1-L4, day 7). As the *in situ* capture of brood VOC did not allow us to separate brood emissions from potential emissions by larval food, our results suggest that the presence of 2-heptanone in our experiment during the early larval stages may be explained by the presence of the larval food in the brood cells. This would indicate that this compound is released from the larval food, even if we cannot rule out a possible emission by larvae.

The compound 1-hexanol (s09), isoamyl acetate (s10) and 1-octanol (s32) are synthetized in the Koschevnikov gland, close to the stinger [50, 54], and are known to being part of the alarm pheromone of adult workers [3, 54, 59]. The main functions of the alarm pheromone for colony defence are increased flight activity, recruitment of individuals and behavioural responses to moving targets. Not all active chemical compounds trigger these three functions. The common function of isoamyl acetate, 1-hexanol and 1-octanol is their ability to recruit other adults [54]. In our experiment, 1-hexanol (s09) had its emission peak at the 5th larval stage (L5, day 8) and remained abundant at the time of operculation (Capping, day 9) and at the last pupal stages (SP, days 17–18). The compound 1-octanol (s32) was captured mainly on days 8, 9 and 10 (L5, Capping, PP), with a peak at the time of operculation. Due to the synchronization between the emission of these compounds and the capping behaviour observed during the experiment, we hypothesise that 1-hexanol and 1-octanol emission by the brood may play a role in eliciting capping behaviour by workers. This hypothesis needs to be validated by behavioural assays, such as those already used to confirm the role of brood pheromone compounds as a capping signal [7].

The amount of isoamyl acetate in the Koschevnikov gland differs according to the age of the worker. Nurse bees displayed more isoamyl acetate in their gland than guards or foragers, suggesting another function in a context other than the defence of the colony [62]. This compound had already been reported as part of VOC of uncapped brood frames in the presence of adult workers [25]. In our study, we captured isoamyl acetate (s10) mainly during the larval stages (L1-L4). This suggests that broods may emit isoamyl acetate as a chemical signal for care to the nurse bees. This signal can have various meanings such as a signal that the brood is healthy, a call for feeding, for temperature or humidity control. Behavioural assays are needed to validate this hypothesis.

### Compounds emitted by the brood but not described as pheromones

In this study, we found 32 compounds characterising the brood belonging to six chemical families, including ten alcohols (prenyl alcohol (s01), 2,3-butanediol (s02), 2,3-butanediol (s03), 1-hexanol (s09), 1-heptanol (s17), 1-octanol (s32), (E)-linalool furanoxide (s35), linalool (s38), phenylethyl alcohol (s41), (E)-linalool pyranoxide (s45)), one aldehyde (phenylacetaldehyde (s28)), seven alkanes (octane (s05), nonane (s13), tridecane (s54), pentadecane (s65), heptadecane (s71), nonadecane (s74), eicosane (s75)), five esters (isoamyl acetate (s10), hexenyl acetate (s24), methyl benzoate (s37), methyl salicylate (s47), γ-octalactone (s52)), two ketones (2-heptanone (s12), 2-nonanone (s36)) and seven terpenes (α-pinene (s15), β-pinene (s18), (Z)-β-ocimene (s27), α-ocimene (s29), (E)-β-ocimene (s30), allo-ocimene (s42), nonadecene (s73)). As previously mentioned, only five compounds have been described as pheromonal compounds in bees. Within the remaining 27 compounds, 13 have already been described in brood, either as being emitted by the brood (octane (s05), nonane (s13), α-pinene (s15), (E)-linalool furanoxide (s35), 2-nonanone (s36), linalool (s38), methyl benzoate (s37), allo-ocimene (s42), methyl salicylate (s47), γ-octalactone (s52), tridecane (s54), heptadecane (s71), nonadecane (s74), eicosane (s75)) [19, 25, 26, 40, 51, 52, 55], or by dead brood extracts (octane (s05), nonane (s13), eicosane (s75) tridecane (s54)) [41]. Our results are in accordance with these studies. The 14 other compounds found to characterize the brood (prenyl alcohol (s01), 2,3-butanediol (s02), 2,3-butanediol (s03), 1-heptanol (s17), β-pinene (s18), hexenyl acetate (s24), (Z)-β-ocimene (s27), phenylacetaldehyde (s28), α-ocimene (s29), phenylethyl alcohol (s41), allo-ocimene (s42), (E)-linalool pyranoxide (s45), pentadecane (s65), nonadecene (s73)) have already been described as being present in the VOC of hive products (honey, propolis, larval jelly, pollen) or floral extracts [44–47, 55, 63, 64], but we described them in honey bee brood for the first time. Nevertheless, α-pinene (s15) and β-pinene (s18) have been described as part of the odours of termite guardians [65] or as being present in Dufour’s gland of ants [66]. The presence of VOC previously measured in hive products in brood emissions is not surprising as hive products are involved in nutrition (honey, larval jelly and pollen) and protection (honey, propolis, larval jelly) against various pathogenic agents of all the individuals in the colony [33, 67–71]. These chemical compounds could be brought to the uncapped brood cells by nurses when they visit brood cells during caring behaviours. Then, compounds can be trapped in and emitted from the wax of brood cells, or released at specific times by the brood as a means of communication with adults.

### Egg compounds

We found that eggs emitted a blend of five VOC: α-pinene (s15), β-pinene (s18), (E)-β-ocimene (s30), methyl benzoate (s37) and γ-octalactone (s52). Even if all of these compounds are maximally abundant at stages of brood development other than the egg stage, this study is the first to describe these 5 compounds as characteristic of eggs. The compound β-ocimene (s30) and methyl benzoate (s37) have already been found on VOC from frames containing eggs and one-day-old larvae [25]. Here, we confirm the presence of (E)-β-ocimene and methyl benzoate in egg brood cells and suggest that (E)-β-ocimene and methyl benzoate take part in the egg odour blend. Heptadecane (s71) had already been found in VOC from frames containing fourth and fifth instar larvae [26]. We found heptadecane on the first day of eggs. Here, we suggest that heptadecane takes part in the newly laid egg odour blend. α-pinene (s15) and β-pinene (s18) can be found in the VOC hive atmosphere [48]. α-pinene has also been found in VOC of larvae and pupae [26], and in VOC from brood frame with adult workers [25]. β-pinene has been found in VOC of honey [44]. In our study, frames were empty of bees and captures were done outside the hive. These two compounds were higher in brood samples than in control wax samples, thus suggesting that they could be part of the blend of VOC emitted by eggs or by cells polished by the workers for the queen to lay eggs.

Beginning our captures on the first day of the egg stage allowed us to discover that honey bee eggs and healthy larvae emit γ-octalactone (s52). γ-octalactone has been described in *Apis dorsata* as an alarm pheromone [72], and was recently described for the first time in *A*. *mellifera* larvae [19]. The authors found that the amount of γ-octalactone increased with the infection by European foulbrood (*M*. *plutonius*). This compound has also been described as stimulating oviposition in flies [73, 74], and as being present in the metapleural glands of ants [66], glands that allow for the marking of the nest [75]. Both of these observations corroborate our observation of γ-octalactone in eggs, and suggest that it may also be involved, along the other four compounds, in egg marking by the queen or polished cell marking by the workers. A comparison between queen and worker eggs would be interesting to solve this question.

### Description of brood VOC emissions at key moments of development

Chemical compounds described in this study had their maximum emission at different times of brood development. Nevertheless, most of these compounds had their peaks during the capping and capped larval stage of the brood (day 9 to 10: Capping, SL).

Larvae have a constant need to communicate with workers in order to be fed. The modulation of the pheromone signal of larvae during their development allows an adaptation of the feeding behaviour of the worker bees [7, 9, 27]. Here, four compounds had their maximum emission at the L1-L4 stage (isoamyl acetate (s10), 2-heptanone (s12), γ-octalactone (s52) and methyl salicylate (s47)) and two at the L5 stage (1-hexanol (s09) and (E)-linalool furanoxide (s35)). The modulation of VOC emitted between the L1-L4 stage and the L5 stage could allow for modulation of worker feeding behaviour, but perhaps more importantly signal the need for capping preparation.

The capping of brood cells is a key moment in brood development. Workers close the brood cell around the 9^th^ day after egg laying. The brood is no longer fed by workers, but the larvae feed on the jelly left on the bottom of the cell and then start to weave their cocoon and prepare for metamorphosis [6]. It has already been shown that the BEP, and particularly methyl esters present in this pheromone, trigger this capping behaviour [7]. Here, we found 11 compounds at their maximum at the time of brood capping (day 9) (prenyl alcohol (s1), 2.3-butanediol (s02 and s03), octane (s05), nonane (s13), α-pinene (s15), 2-nonanone (s36), phenylethyl alcohol (s41), heptadecane (s71), nonadecane (s74), eicosane (s75)). A complex mixture of VOC and BEP could trigger the capping of brood cells by workers. More research is necessary to determine if, and which of, these compounds alone trigger capping behaviour. The complexity of the mixture captured at this key day in brood development opens up new perspectives in understanding chemical communication between brood and adults.

After capping, the larva stretches out and starts weaving its cocoon (day 11, SL) [6]. At this time, we found seven compounds at their maximum emission (phenylacetaldehyde (s28), (E)-β-ocimene (s30), 1-octanol (s32), linalool (s38), allo-ocimene (s42), tridecane (s54) and pentadecane (s65)). Then the larva starts its metamorphosis (day 12, PP) [6]; we found three compounds at their maximum release on this day (hexenyl acetate (s24), (Z)-β-ocimene (s27), α-ocimene (s29)). Communicating at the beginning of metamorphosis could allow the workers to monitor the health of the brood. A modification of those compounds emitted by the brood just after capping, for example because of *Varroa* or *Nosema* parasitism, may trigger the cleaning behaviour by the adults [16, 19, 76].

The pupal stages were characterised by the peaking of two terpene compounds, one during NSP, and one during SP. β-pinene (s18) had its maximum emission in the early pupal stages (NSP), mainly on days 14 to 16. Nonadecene (s73) is the only compound that had its peak of emission during the last stage of brood development (SP), especially during the last 3 days before adult emergence. At the end of metamorphosis, the brood is not fed and not subject to hygienic behaviour [6]. Here, we described for the first time an emission of VOC from the brood before the emergence of the adult bee. When it could just be part of the chemical process of development and given off as a by-product, it also suggests a potential emergence signal, with a possible role in the acceptance of newly emerged workers by adults.

Interestingly, the emission of chemical families correlates with the age of the brood. Uncapped broods emitted mainly ketones, esters and alcohols, while capped broods emitted alkanes and terpenes. The emission of volatile esters (isoamyl acetate (s10), methyl benzoate (s37), methyl salicylate (s47), γ-octalactone (s52)) in larval stages is consistent with the emission of BEP from the brood at these stages [7]. These compounds could be complementary to the BEP. In addition, the brood emitted several highly volatile alkanes. Adults recognise their kin with cuticular hydrocarbon mixtures of low volatile alkanes and alkenes [77, 78]. It has been shown that long carbon chain alkanes are differentially recognised by adult honey bees [79], and it would be interesting to see if short carbon chain alkanes are also involved in nestmate recognition.

This study is the first study of VOC emissions of brood covering the entire honey bee developmental cycle, from egg laying to adult emergence. Here, we show the complexity of the volatile chemical messages emitted by the brood at different developmental stages. When Martin *et al*. [80] suggested low volatile compound as egg marking signal, we describe the olfactory signature made of volatile compounds of *A*. *mellifera* eggs that could also have a potential implication in the marking of queen eggs. We also show a potential emergence signal. While the compounds identified could have a potential involvement in the chemical recognition of brood and its needs by the workers, some of the compounds identified could also be the result of metabolic by-products. Our suggestions need to be assessed using appropriate biological tests. This study opens up new perspectives on the intricate relationship between brood and adult bees, and the behaviour of nurse bees towards the brood. Brood emission of molecules already described as part of chemical communication in the adult reinforces the idea that chemical communication in the bee is highly context-dependent and based on the quantity of each molecule emitted.

## Supporting information

S1 Fig **Redundancy analysis (RDA) performed on chemical compounds captured on brood and control samples (A) for “Eggs” group (day 1 to day 3), (B) “Sclerotised Pupae” group (day 17 to day 21).** For each day: brood sample n = 9, control sample n = 9. Points represent samples (blue: brood, red: wax). Compounds are represented by their IDs and grey arrows. The compounds characterising the brood that are retained for the rest of the analysis are shown in bold.
(TIF)

S2 Fig Representative gas chromatography profiles of each brood stage. The compounds listed are those identified as brood specific. The other peaks represent fibre peaks or peaks specific to the wax control. (a) Eggs, (b) L1-L4: 1^st^ to 4^th^ instar larvae, (c) L5: 5^th^ instar larvae, (d) Capping: adult capping behaviour, (e) LS: larvae spinning cocoon, (f) PP: pre-pupae, (g) NSP: non-sclerotised pupae, (h) SP: sclerotised pupae. °: Unsaturation remains unknown, *: Stereochemistry remains unknown.
(TIF)

S3 Fig Quantification of (E)-β-ocimene emission for an individual from the 1st instar larval to the 5th pupal stage. Weight data are from previous experiments. Line represents mean values and bars represent standard deviation. For each day, n = 9.
(TIF)

S1 Table Peak areas of the 78 compounds found for each group (brood or wax), at each day and for each colony. (CSV)
