# Supplementary figures and images for: Detailed chemical analysis of honey bee (Apis mellifera) worker brood volatile profile from egg to emergence
Source: PLoS One. 2023 Feb 21;18(2):e0282120. doi: 10.1371/journal.pone.0282120 (PMC9943000; doi:10.1371/journal.pone.0282120)

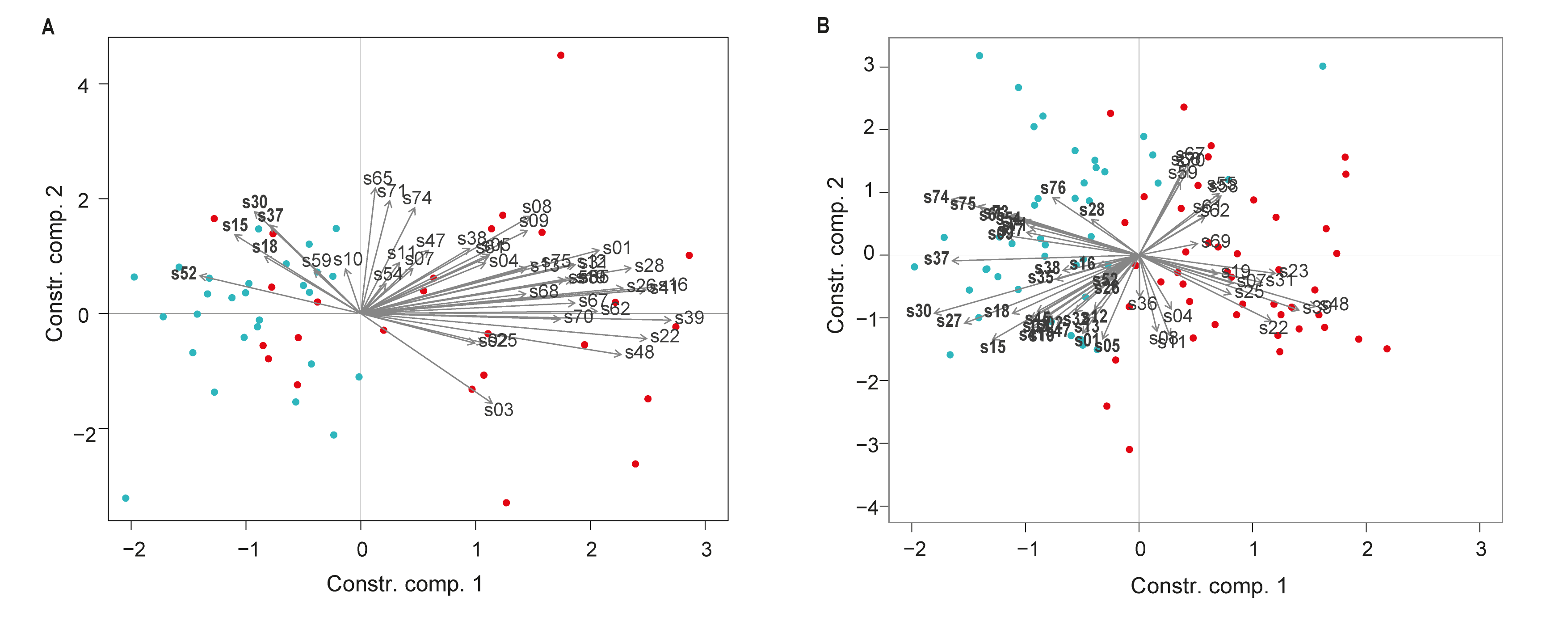

Supplement: S1 Fig — Redundancy analysis (RDA) performed on chemical compounds captured on brood and control samples (A) for “Eggs” group (day 1 to day 3), (B) “Sclerotised Pupae” group (day 17 to day 21). For each day: brood sample n = 9, control sample n = 9. Points represent samples (blue: brood, red: wax). Compounds are represented by their IDs and grey arrows. The compounds characterising the brood that are retained for the rest of the analysis are shown in bold. (TIF) [file pone.0282120.s001.tif]

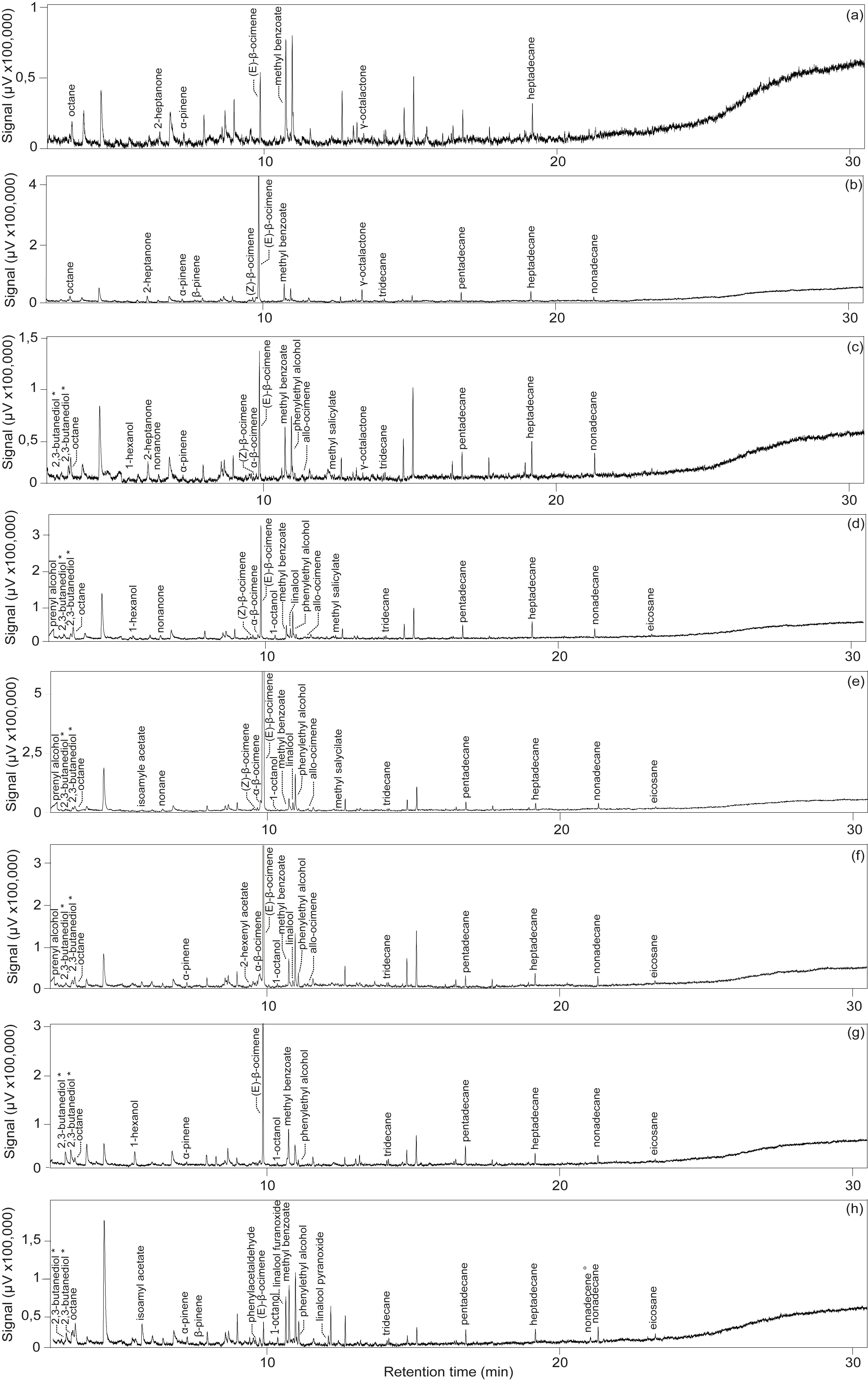

Supplement: S2 Fig — The compounds listed are those identified as brood specific. The other peaks represent fibre peaks or peaks specific to the wax control. (a) Eggs, (b) L1-L4: 1st to 4th instar larvae, (c) L5: 5th instar larvae, (d) Capping: adult capping behaviour, (e) LS: larvae spinning cocoon, (f) PP: pre-pupae, (g) NSP: non-sclerotised pupae, (h) SP: sclerotised pupae. °: Unsaturation remains unknown, *: Stereochemistry remains unknown. (TIF) [file pone.0282120.s002.tif]

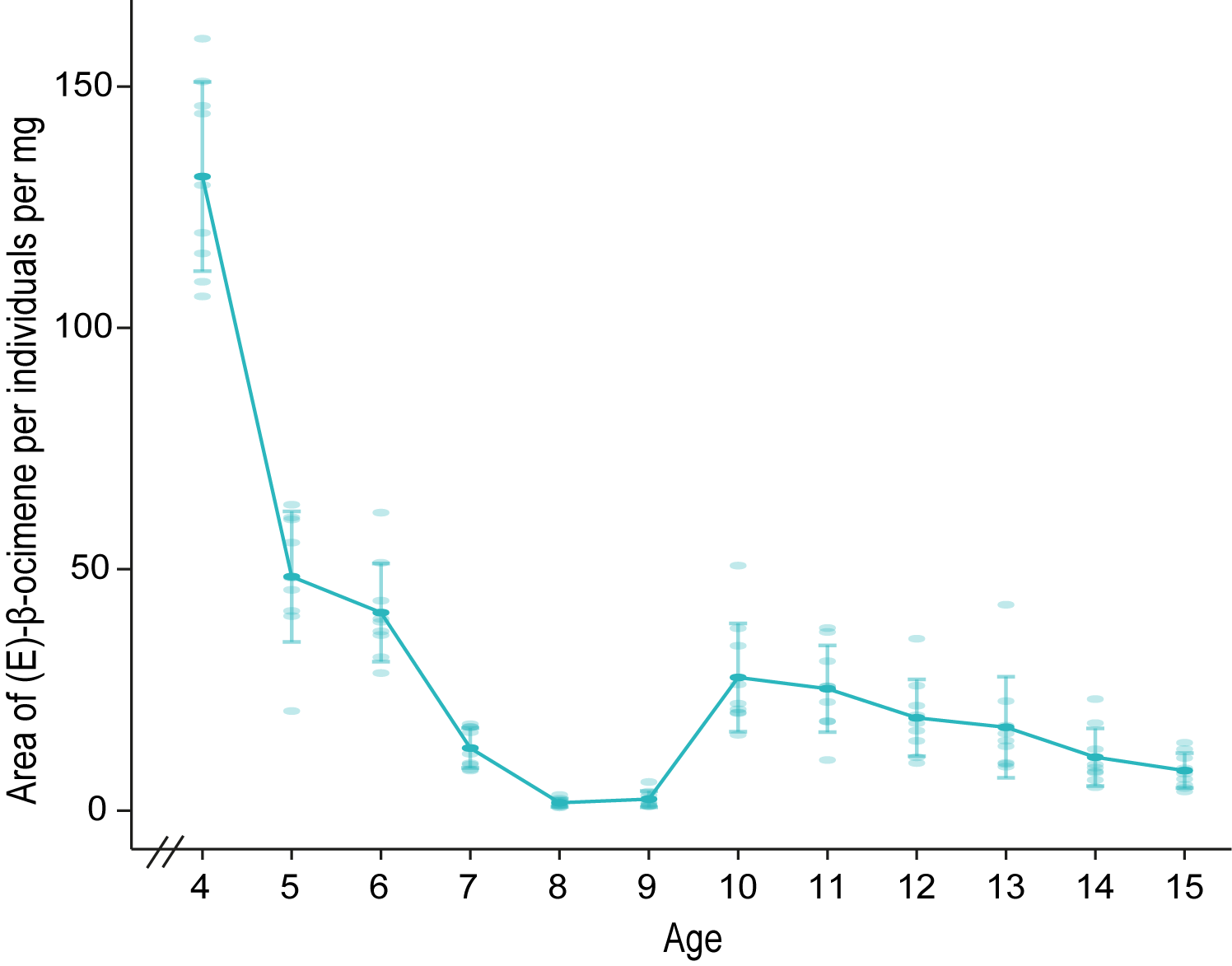

Supplement: S3 Fig — Weight data are from previous experiments. Line represents mean values and bars represent standard deviation. For each day, n = 9. (TIF) [file pone.0282120.s003.tif]
